# Potential role of the *HOXD8* transcription factor in cisplatin resistance and tumour metastasis in advanced epithelial ovarian cancer

**DOI:** 10.1038/s41598-018-31030-3

**Published:** 2018-09-07

**Authors:** PengMing Sun, YiYi Song, DaBin Liu, GuiFen Liu, XiaoDan Mao, BinHua Dong, Elena Ioana Braicu, Jalid Sehouli

**Affiliations:** 1 Laboratory of Gynaecologic Oncology, Fujian Provincial Maternity and Children’s Hospital, Affiliated Hospital of Fujian Medical University, No 18. Dao Shan Road, 350001 Fuzhou, Fujian Province P.R. China; 2 Department of Gynaecology, Fujian Provincial Maternity and Children’s Hospital, Affiliated Hospital of Fujian Medical University, No 18. Dao Shan Road, 350001 Fuzhou, Fujian Province P.R. China; 3 Department of Gynaecologic Oncology and Gynaecology, Charité/Campus Virchow-Klinikum, European Competence Centre for Ovarian Cancer University of Berlin, 13353 Berlin, Germany

## Abstract

Few studies have examined the potential transcription factor (TF) simultaneously associated with cisplatin resistance and metastasis in ovarian cancer. To assess a related mechanism, a 345-channel protein/DNA array and transcriptional activity ELISA were performed to compare the TF activities in the cisplatin-sensitive SKOV3 and cisplatin-resistant SKOV3-DDP cells and in HO-8910 and the homologous highly metastatic HO-8910PM cells. In SKOV3-DDP *vs*. SKOV3 cells, 43 TFs were up-regulated, while 31 were down-regulated. In HO-8910PM *vs*. HO-8910 cells, 13 TFs were up-regulated, while 18 were down-regulated. In these two models, 4 TFs (*HOXD8(1), HOXD8(2), RB, RFX1/2/3*) were simultaneously up-regulated, and 9 TFs (*SRE, FKHR, Angiotensinogen ANG-IRE, Pax2, CD28RC/NF-IL2B, HLF, CPE, CBFB and c-Ets-1*) were down-regulated. *HOXD8* mRNA and protein expression levels measured by reverse transcription polymerase chain reaction and ELISA, respectively, were significantly higher in SKOV3-DDP and HO-8910PM than in their corresponding cell lines (both *p* < 0.05). In 52 cases of different ovarian disease, the patients with recurrent and cisplatin-resistant ovarian cancer had higher expression levels of HOXD8 than patients with primary malignant tumours (*p* = 0.018, *p* = 0.001) or benign tumours (*p* = 0.001, *p* < 0.001). Taken together, these results suggest that *HOXD8* is potentially associated with both cisplatin resistance and metastasis in advanced ovarian cancer.

## Introduction

Ovarian cancer is a deadly disease that affects women globally. The worldwide incidence of ovarian cancer is currently at 225,500 new diagnoses each year^1,2^. The majority of ovarian cancers are diagnosed at an advanced stage when they have already metastasized to other organs outside the pelvis, mostly because of a lack of characteristic symptoms, a lack of effective early-screening strategies, and their aggressive tumour behaviour^3,4^. These factors lead to the high mortality rate of ovarian cancer. A deeper understanding of the molecular mechanisms that regulate ovarian cancer growth and tumour progression is needed.

High-grade ovarian cancers generally grow rapidly, metastasize early, and have a very aggressive disease course with a high rate of chemotherapy resistance^4^. Thus, ovarian cancer invasion and metastasis still represent major hurdles that must be overcome to improve patient outcomes. Over the course of several decades, a number of chemotherapeutic agents that target DNA and microtubule structures have been developed for treating ovarian cancer^5^. However, the majority of women with advanced stage ovarian cancer are only temporarily chemotherapy-sensitive and experience relapse within the first three years after primary diagnosis. Thus, further study of chemotherapy resistance mechanisms is critical for improving the clinical outcomes of patients with advanced ovarian cancer.

Occasionally, metastasis and resistance may occur during or immediately after the application of chemotherapy. These outcomes occur through a series of processes that are closely associated with different genes. It has been reported that several genes are involved in the chemotherapy resistance pathway with a high degree of interaction. The gene chip technique has been widely used to detect differences in gene expression. This technique is advantageous compared to the traditional methods by which differences in only a single or several genes can be observed^6^. However, to date, there are few studies that confirm the common mechanisms of resistance and metastasis in ovarian cancer^7–10^.

Eukaryotic gene expression is regulated by proteins called transcription factors, which bind to the promoter region of a gene^11^. Transcription factors facilitate the binding of RNA polymerase, which initiates the expression of the gene^12^. The expression or activity of transcription factors may be regulated in a cell-specific, tissue-specific, or cell cycle-dependent manner. Regulation can also be mediated by interactions with other proteins. Through different combinations of these regulatory mechanisms, eukaryotes are able to elicit myriad gene expression patterns^13^. Analysing transcription factor activity is critical in developing a thorough understanding of how gene expression is regulated. Homeobox (*HOX)* genes, a highly conserved subgroup of the homeobox superfamily, have crucial roles in development, regulating numerous processes, including cell division, adhesion, proliferation, apoptosis, and differentiation, during development and normal cellular processes. Aberrations in *HOX* gene expression have been reported in abnormal development and malignancy, indicating that altered expression of *HOX* genes could be important for both oncogenesis and tumour suppression^14,15^. Therefore, *HOX* gene expression could be important in diagnosis and therapy.

In the present study, we used a high-throughput DNA-protein microarray to analyse potential transcription factors associated with ovarian cancer cisplatin-resistance and metastasis. To the best of our knowledge, this report is the first to describe the association between overexpression of *HOXD8* and cisplatin-resistance and metastasis in ovarian cancer cells.

## Results

### Different biological behaviours of two pairs of cellular models *in vitro*

**Figure 1:**
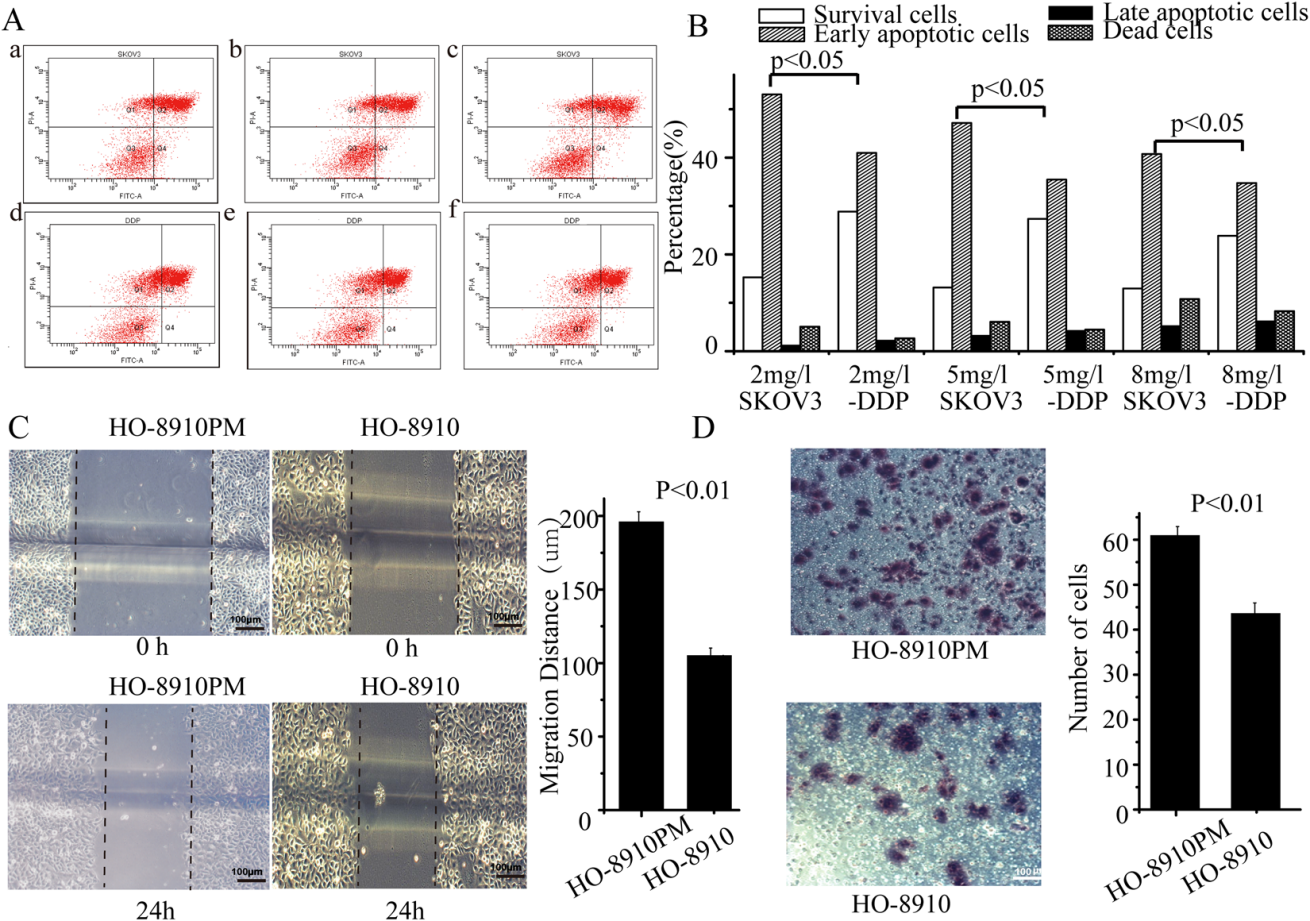
Different biological behaviours in two pairs of ovarian cancer cell lines. In flow cytometry (**A**), a–c were SKOV3 cell lines and d–f are SKOV3-DDP cell lines treated with 2, 5, or 8 mg/l of cisplatin. Q1, Q2, Q3, Q4 represent dead cells, late apoptotic cells, survival cells, and early apoptotic cells, respectively. At different concentrations of 2, 5, or 8 mg/l, SKOV3 and SKOV3-DDP cells have significant differences in cisplatin resistance (*p* < 0.05) (**B**). For HO-8910PM and HO-8910 cell lines, pictures were taken of the cells at 0 h, then incubated overnight, and photographed again. Cell migration distance in the HO-8910 cell line was less than that in the HO-8910PM cell line (p < 0.01) (**C**). Cells were incubated on migration wells for 48 h, the number of cells that migrated to the lower side of the filter were counted (**D**). The results are shown as the mean ± SD (*p* < 0.01 compared to the HO-8910 cell line).

When treated with 2, 5, or 8 µg/ml cisplatin, SKOV3 cells had significantly lower cell survival than SKOV3-DDP cells (15.10% *vs* 28.70%, *p* < 0.05, 13.00% *vs* 27.20%, *p* < 0.05, 12.80% *vs* 23.70%, *p* < 0.05, respectively) (Fig. 1A,B). The results of the cell migration assay showed that the migration distances of the HO-8910PM and HO-8910 cell lines at 24 h were significantly different with distances of 196.12 ± 6.73 µm and 105.32 ± 4.88 µm, respectively (*t* = 18.90, *p* = 0.01) (Fig. 1C). The difference between the number of cells permeating the septum in the HO-8910PM group (61.00 ± 1.91 cells) and the HO-8910 group (43.67 ± 2.27 cells) was statistically significant (*t* = 26.95, *p* < 0.01) (Fig. 1D).

### Protein/DNA Arrays of different transcription factors

**Figure 2:**
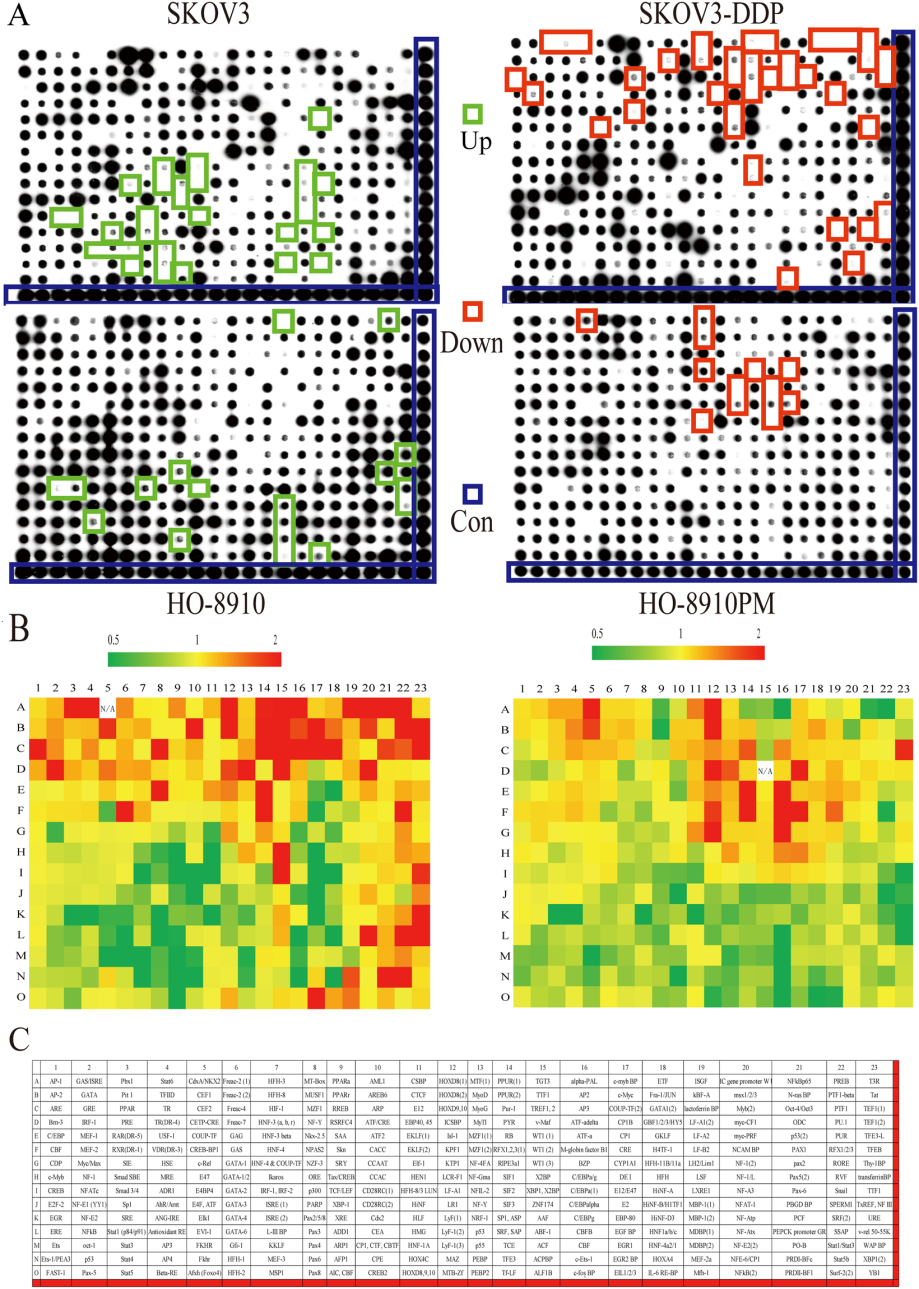
Different activity of transcription factors in protein/DNA arrays. As seen on the X-ray film (**A**), some transcription factor activities were up-regulated (red boxes) and some were down-regulated (green boxes) in two pairs of ovarian cancer cells. Three hundred forty-five transcription factors were observed (**B**). A heat map of SKOV3-DDP/SKOV3 (left) or HO-8910PM/HO-8910 (right) of different transcription factors (**C**). If the colour was closer to red, the transcription factor activity was higher. In contrast, the colour closer to green indicated lower activity. N/A meant there were no transcription factor activities in HO-8910 or SKOV3 cell lines.

**Figure 3:**
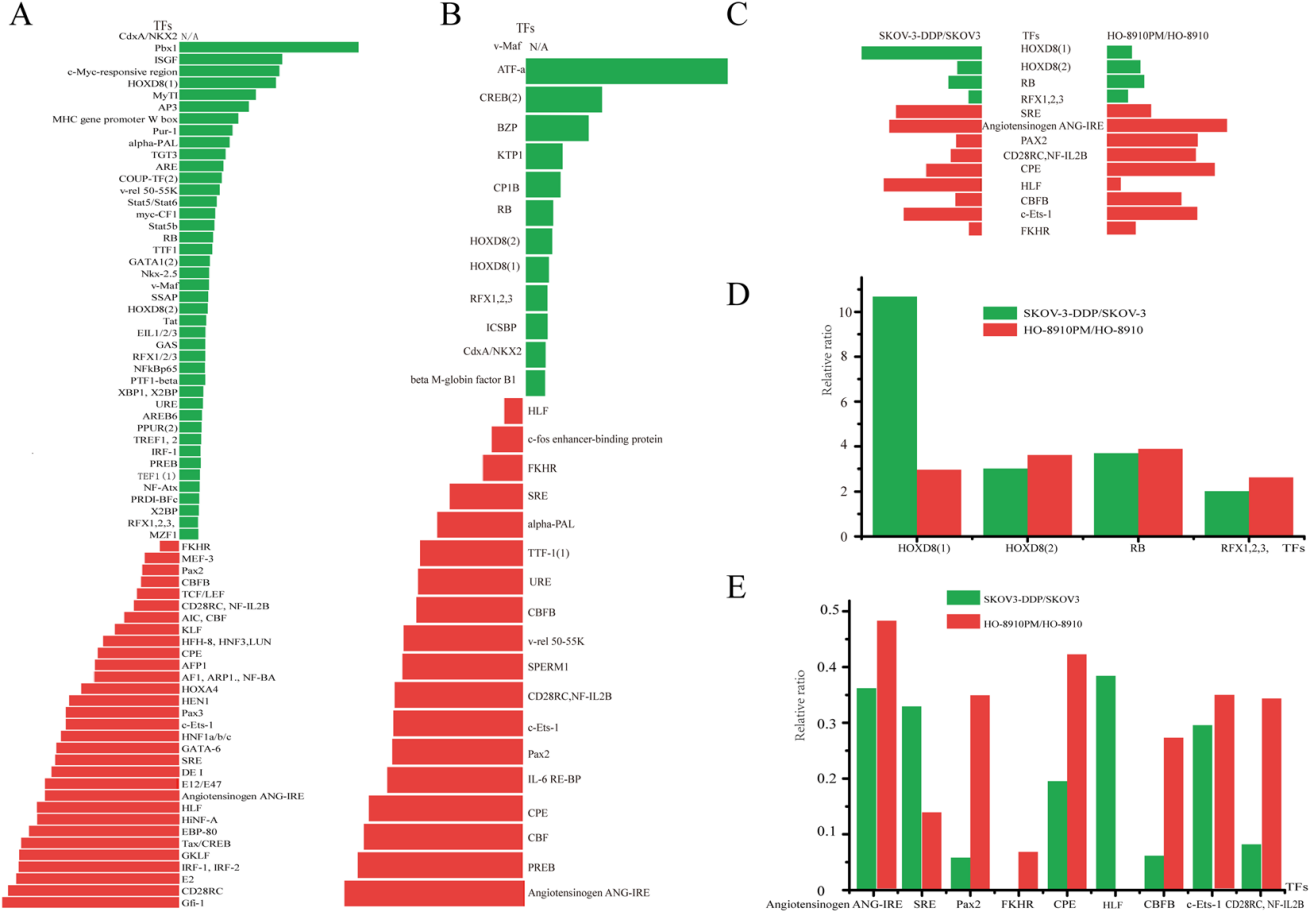
Analysis of protein/DNA arrays. Using the expression in SKOV3 and HO-8910 cells as a benchmark, differential expression of transcription factors was observed in the SKOV3-DDP/SKOV3 or HO-8910PM/HO-8910, increased or decreased by two-fold, respectively. Forty-three transcription factors were up-regulated (green) and thirty-one down-regulated (red) in SKOV3 and SKOV3-DDP cells (**A**). Thirteen transcription factors were up-regulated (green) and eighteen down-regulated (red) in HO-8910PM and HO-8910PM cells (**B**). In conclusion, four up-regulated (green), and nine down-regulated (red) transcription factors were associated with ovarian cancer cisplatin-resistance and metastasis (**C**). The concrete activities were shown (**D**,**E**).

The results of the transcription factor chip test of the ovarian cancer cell line SKOV3 and its cisplatin-resistant strain and HO-8910 and its highly metastatic cells are shown in Fig. 2A. Of a total 345 candidate transcription factors (Fig. 2B,C), 74 transcription factors were associated with cisplatin-resistance. Compared with the SKOV3 cell line, 43 transcription factors were up-regulated, and 31 were down-regulated in the SKOV3-DDP cell line (Fig. 3A). Compared with the HO-8910 cells, 13 transcription factors were up-regulated and 18 were down-regulated in the HO-8910PM cells (Fig. 3B). Taken together, 13 transcription factors associated with ovarian cancer cisplatin-resistance and metastasis were identified, of which four were up-regulated (*HOXD8(1), HOXD8(2), RB, RFX1/2/3*) and nine down-regulated (*SRE, FKHR*, *Angiotensinogen ANG-IRE, Pax2, CD28RC/NF-IL2B, HLF, CPE, CBFB* and *c-Ets-1*) (Fig. 3C–E). Specific content of TFs as follows in Table 1.

**Table 1:** Detailed information of 13 common TFs.

Transcription Factor	Description	References/Publications
HOXD8(1)	homeobox D8	EMBO J. 10: 4177–4187 (1991).
HOXD8(2)	homeobox D8	EMBO J. 10: 4177–4187 (1991).
RB	retinoblastoma tumour suppressor protein	Mol. Cell. Biol. 12: 4327–4333 (1992).
RFX1,2,3	regulatory factor X (RFX)	Nucleic Acids Res. 19: 6269–6276 (1991).
SRE	SREBF: sterol regulatory element binding transcription factor	MCB. 12:2432-2443 (1992).
Angiotensinogen ANG-IRE	Angiotensinogen(ANG) insulin-response element (IRE)	Endocrinology. 142: 2577–85 (2001).
Pax2	paired box gene 2(gene5/gene8)	BBRC. 266: 532–541 (1999).
CD28RC, NF-IL2B	T-cell accessory molecule CD28	Science. 251: 313–316 (1991).
CPE	cap proximal element	J. Biol. Chem. 265: 20524–20532 (1990).
HLF	hepatic leukaemia factor	Mol. Cell. Biol. 14: 5986–5996 (1994).
CBFB	CCAAT-binding factor	J. Biol. Chem. 270: 468–475 (1995).
c-Ets-1	c-Ets-1A; p54c-Ets-1; p54; Ets1; c-Ets-1 54	J. Biol. Chem. 269: 14946–14950 (1994).
FKHR	forkhead box O1A (rhabdomyosarcoma) human	Eur. J. Immunol. 30: 2980–2990 (2000).

### Different transcriptional activity of the same transcription factor in two cell lines

**Figure 4:**
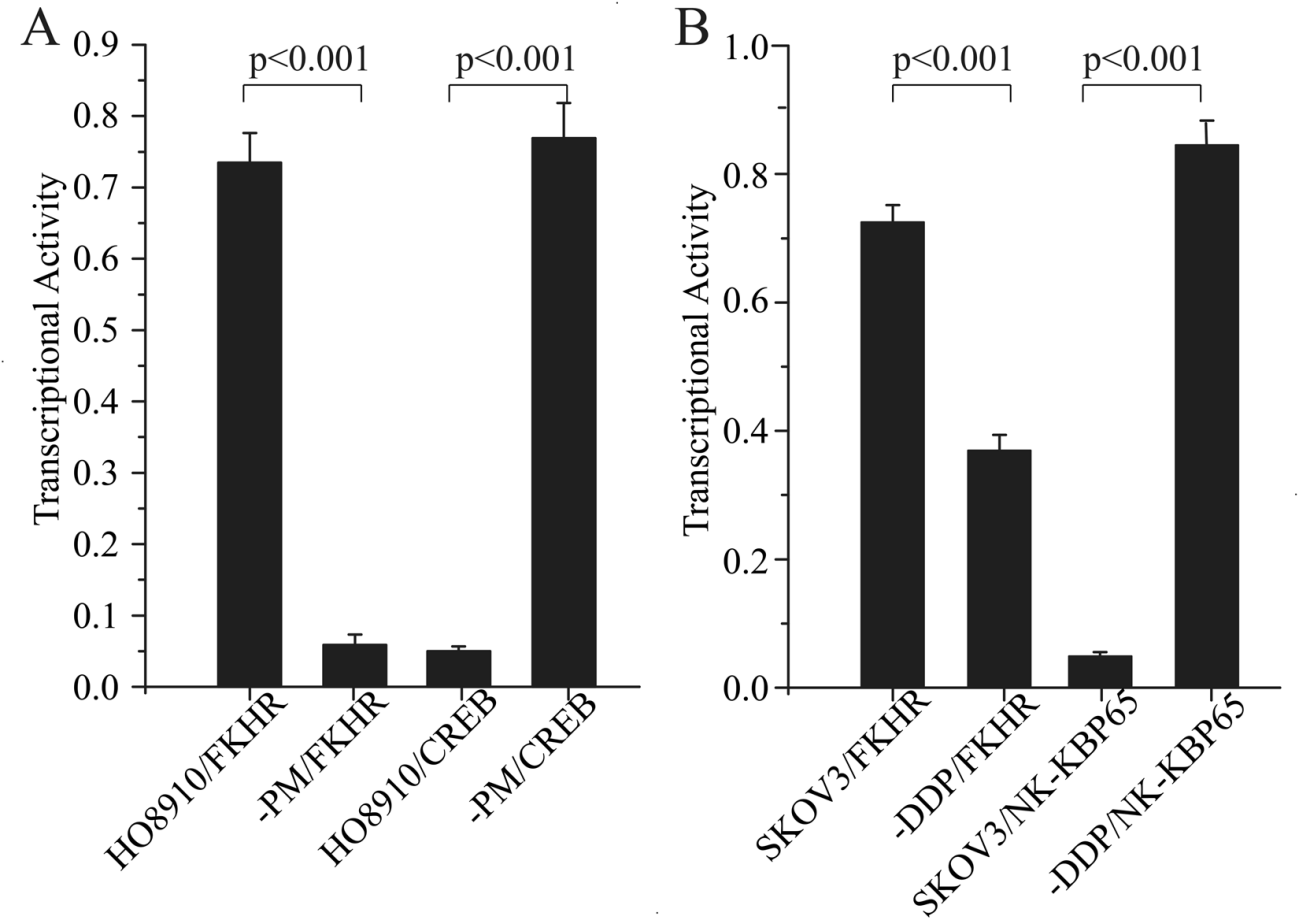
Transcriptional activities of FKHR, CREB and NF-kB P65 were detected through an ELISA-based transcriptional assay in HO-8910PM/HO-8910 cells (**A**) or SKOV3-DDP/SKOV3 (**B**). The transcriptional activity of FKHR in all cell lines is similar to that in the protein/DNA array.

To confirm the results from the protein/DNA array, transcriptional activities of FKHR, CREB and NF-ĸB P65 were detected through an ELISA-based transcriptional assay. The FKHR and CREB transcriptional activities in HO-8910 compared to HO-8910PM cells were 0.73 ± 0.06 *vs*. 0.04 ± 0.01 (F = 12.94, *P* < 0.001), 0.05 ± 0.01 *vs*. 0.77 ± 0.05 pg/ml (F = 3.90, *p* < 0.001), respectively (Fig. 4A). The FKHR and NF-ĸB P65 transcriptional activities were 0.73 ± 0.03 *vs*. 0.37 ± 0.02 (F = 0.06, *p* < 0.001), 0.05 ± 0.01 *vs*. 0.85 ± 0.03 pg/ml (F = 10.35, *p* < 0.001) in SKOV3 and SKOV3-DDP cell respectively (Fig. 4B). The transcriptional activities of FKHR in SKOV3-DDP and HO-8910PM were lower than SKOV3 and HO-8910 cells (p < 0.001, respectively). The results of the ELISA-based transcriptional activity assay were similar to the result from the protein/DNA array.

### Differential expression of *HOXD8* in cell lines

**Figure 5:**
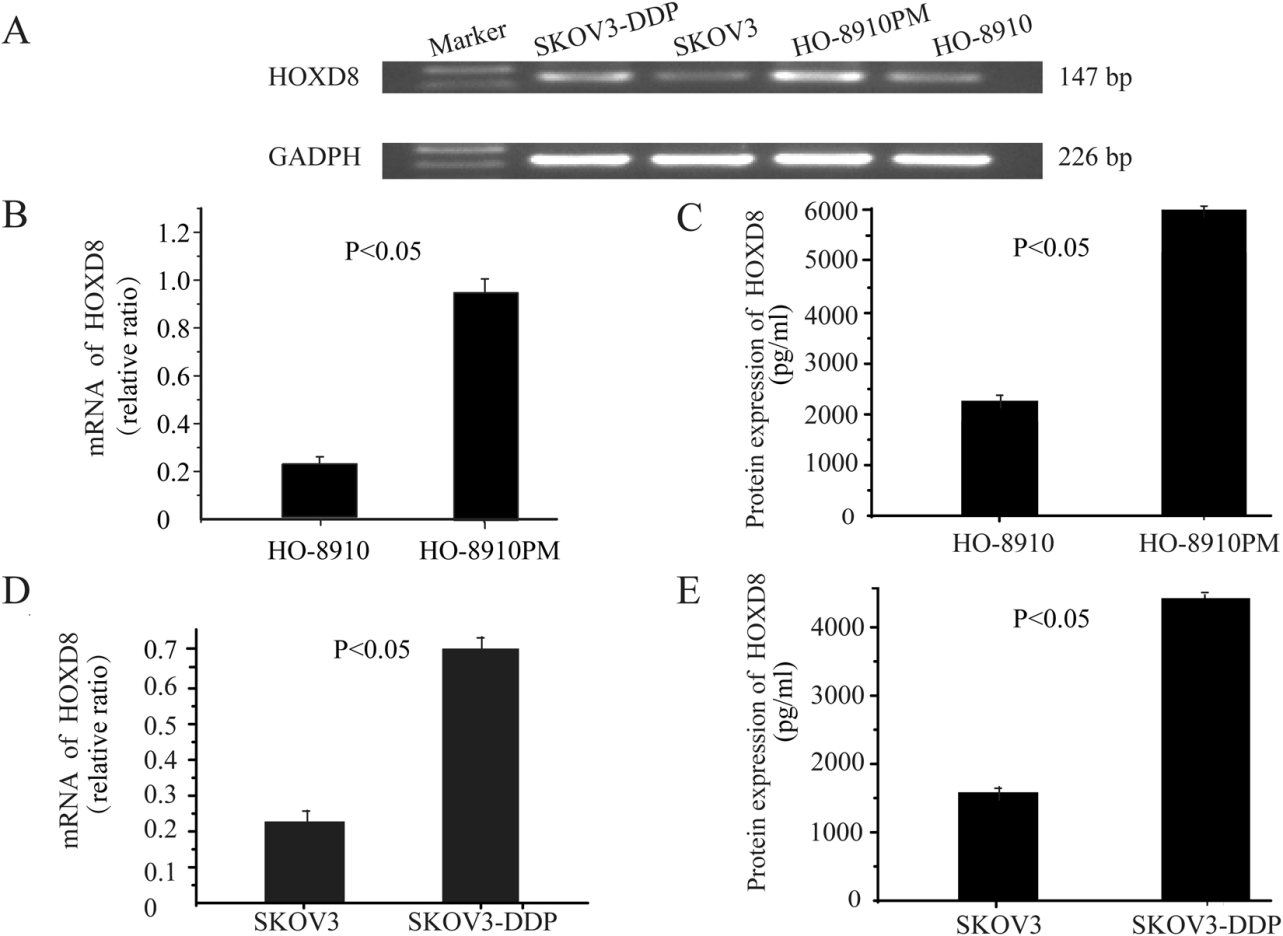
Different expression of HOXD8 in different cell lines (**A**). The mRNA expression levels of *HOXD8* in HO-8910PM cell, HO-8910 cell (0.98 ± 0.08 *vs*. 0.21 ± 0.01, *p* = 0.04) (**B**). ELISA showed that the protein expression of HOXD8 in the HO-8910 cell lines was significantly different from that in HO-8910PM cell lines, (5965.34 ± 1.04 pg/ml *vs*. 1930.50 ± 2.50 pg/ml, *p* < 0.05) (**C**). The mRNA expression levels of SKOV3-DDP were higher in SKOV3 cells (0.71 ± 0.03 *vs*. 0.24 ± 0.04, *p* < 0.05) **(D**). ELISA showed that the protein expression of HOXD8 in the SKOV3 cell line was 1584.40 ± 2.08 pg/ml, but in the SKOV3-DDP cell line, it was 4423.30 ± 1.52 pg/ml. The difference was significant (*p* < 0.05) (**E**).

Compared with HO-8910 and SKOV3 cells, the mRNA expression levels of HOXD8 were increased in SKOV3-DDP and HO-8910PM cells (Fig. 5A). Similarly, the mRNA expression level of HOXD8 was increased in HO-8910PM cells compared with HO-8910 cells (0.98 ± 0.08 *vs*. 0.21 ± 0.01, *p* = 0.036) (Fig. 5B). HOXD8 expression was also significantly increased at the protein level in the HO-8910PM cell line in comparison to HO-8910 cells (5965.34 ± 1.04 pg/ml *vs*. 1930.5 ± 2.5 pg/ml, respectively, *p* < 0.05) (Fig. 5C). A similar trend was observed in the SKOV3-DDP and SKOV3 cell lines. The mRNA expression level of HOXD8 in SKOV3-DDP cells was increased compared with SKOV3 cells (0.71 ± 0.03 *vs*. 0.24 ± 0.04, *p* < 0.05) **(**Fig. 5D). The quantitative protein ELISA results revealed the protein expression of HOXD8 in the SKOV3 cell line was 1584.40 ± 2.08 pg/ml compared to 4423.30 ± 1.52 pg/ml in the SKOV3-DDP cell. The protein level was significantly increased in the SKOV3-DDP cell line compared to that in the SKOV3 cell line (*p* < 0.05) (Fig. 5E).

### High expression of HOXD8 in HO-8910PM cells resistant to cisplatin treatment

**Figure 6:**
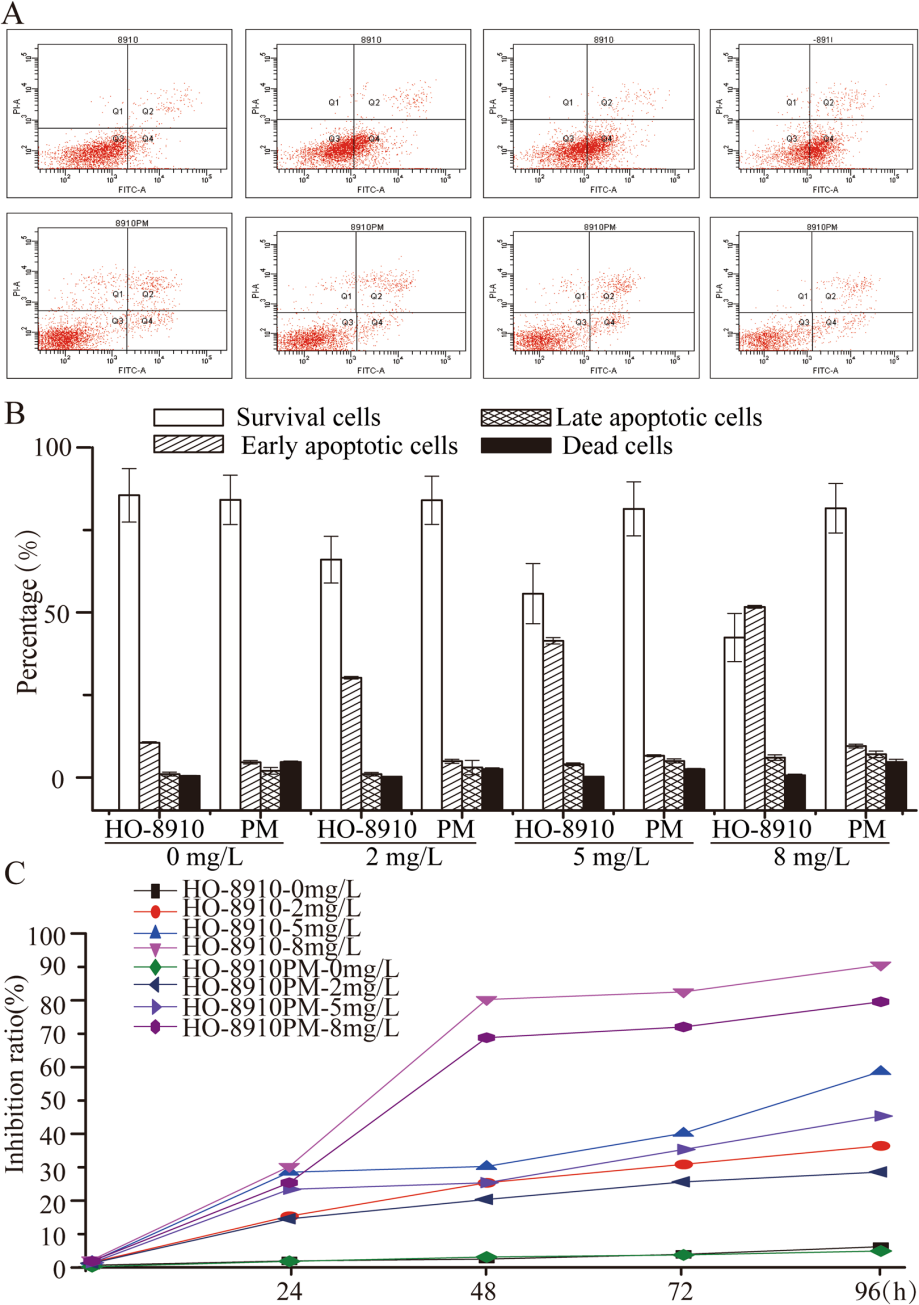
High expression of HOXD8 in HO-8910PM cell line make them cisplatin-resistant. Analysed by flow cytometry (**A**), HO-8910 (upper panel) and HO-8910PM (lower panel) were treated with 0, 2, 5, or 8 mg/L of cisplatin. Q1, Q2, Q3, Q4 represent dead cells, late apoptotic cells, survival cells, and early apoptotic cells, respectively. At different concentrations of 0, 2, 5, or 8 mg/L, these two cells have significant differences in cisplatin resistance (*p* < 0.05) (**B**). Growth curves of HO-8910 and HO-8910PM (**C**).

Since higher expression of HOXD8 was observed in HO-8910PM cells than it in HO-8910 cells, we further compare the response to the treatment with cisplatin in these two cells. Flow cytometry was used to analysed the cellular apoptosis of HO-8910 and HO-8910PM cells treated with 0, 2, 5, or 8 mg/L cisplatin. The results showed HO-8910 cells had significantly lower cell survival than HO-8910PM cells (apoptotic rate in 2 mg/l, 33.50% *vs* 13.4%, *p* < 0.05, in 5 mg/L, 44.00% *vs* 16.10%, *p* < 0.05, in 8 mg/L, 56.80% *vs* 17.90%, *p* < 0.05, respectively) (Fig. 6A,B). MTT assay also revealed that the proliferation of HO-8910 cells was significantly inhibited (Fig. 6C).

### Overexpression of HOXD8 induce the cisplatin-resistance in ovarian cancer

**Figure 7:**
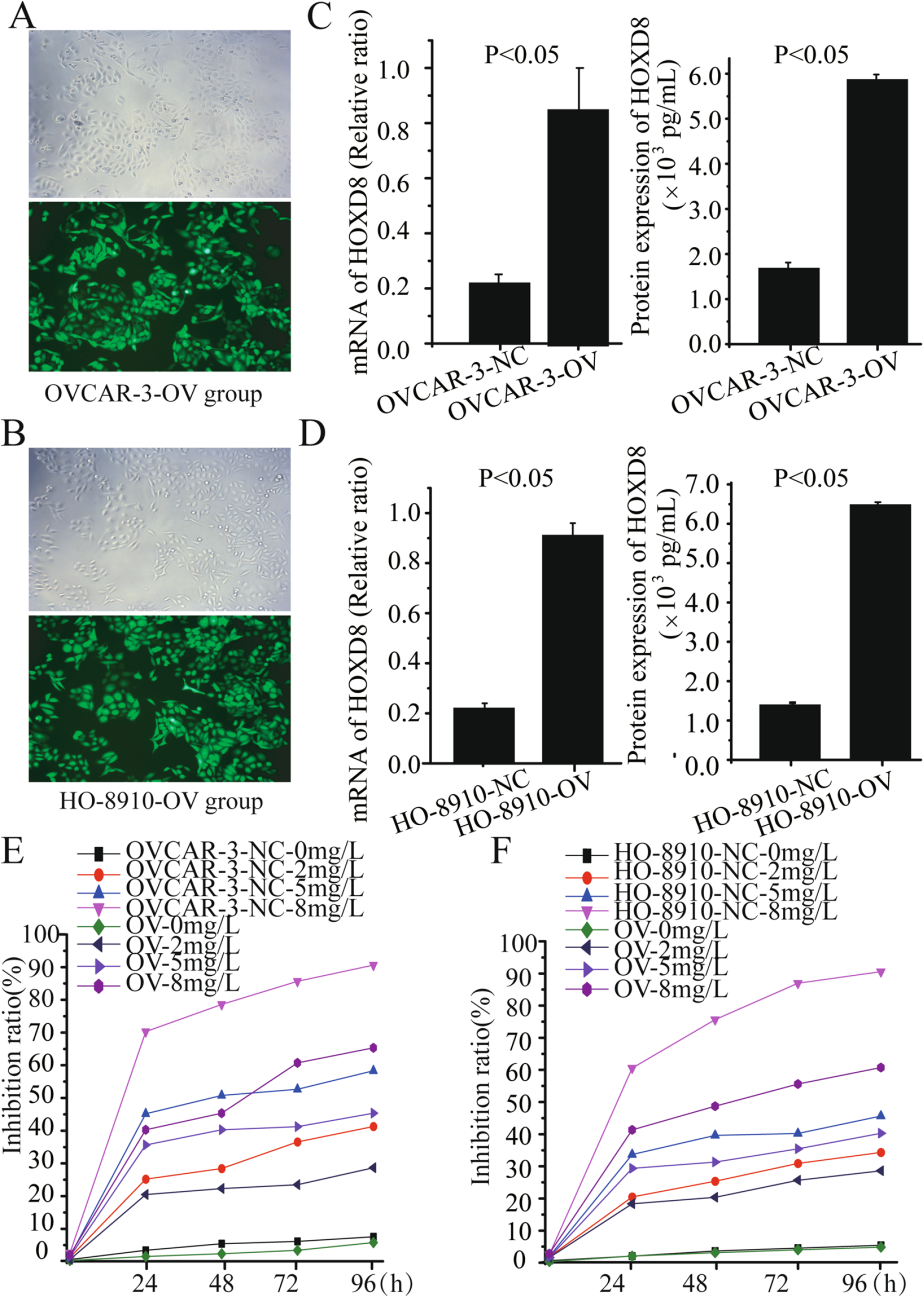
Overexpression of HOXD8 enhancing the cisplatin-resistance in ovarian cancer. After infecting with lentivirus mediated HOXD8 or control vectors (NC group), the ovarian cancer OVCAR-3 and HO-8910 cells were observed by optical microscope and fluorescence microscopic with a magnification of 400 (**A**,**B**). Expression of HOXD8 mRNA and protein were significantly up-regulated in OVCAR-3-OV cell (**C**) and HO-8910-OV cells (**D**). Cellular proliferation of OVCAR-3-NC and OVCAR-3-OV, HO-8910-NC and HO-8910-OV were detected by MTT assay (**E**,**F**).

The lentivirus mediated overexpressing HOXD8 vector was used to infect the OVCAR-3 and HO-8910 cell and names as overexpression group (OV) (Fig. 7A,B). Compared with the OVCAR-3 and HO-8910 cells infected with lentivirus mediated control vector (NC group), the mRNA expression of HOXD8 in OV group was significantly up-regulated in both OVCAR-3-OV (OV 0.85 ± 0.15 *vs*. NC 0.21 ± 0.04, *p* < 0.05) and in HO-8910-OV (OV 0.91 ± 0.05 *vs*. NC 0.22 ± 0.02, *p* < 0.05). Similarly, the quantitative protein ELISA results revealed the significantly up-regulated protein expression of HOXD8 in the OV group (OVCAR-3-OV 0.85 ± 0.15 pg/ml *vs*. NC 0.21 ± 0.04 pg/ml, *p* < 0.05, HO-8910-OV 6523.56 ± 56.78 pg/ml *vs*. NC 1530.52 ± 25.63 pg/ml, *p* < 0.05) (Fig. 7C,D). Results of MTT assay in these two groups also revealed more resistant to the treatment of cisplatin in the HOXD8 overexpresion group than the NC group (Fig. 7E,F).

### HOXD8 expression in the serum of patients with different ovarian diseases

**Figure 8:**
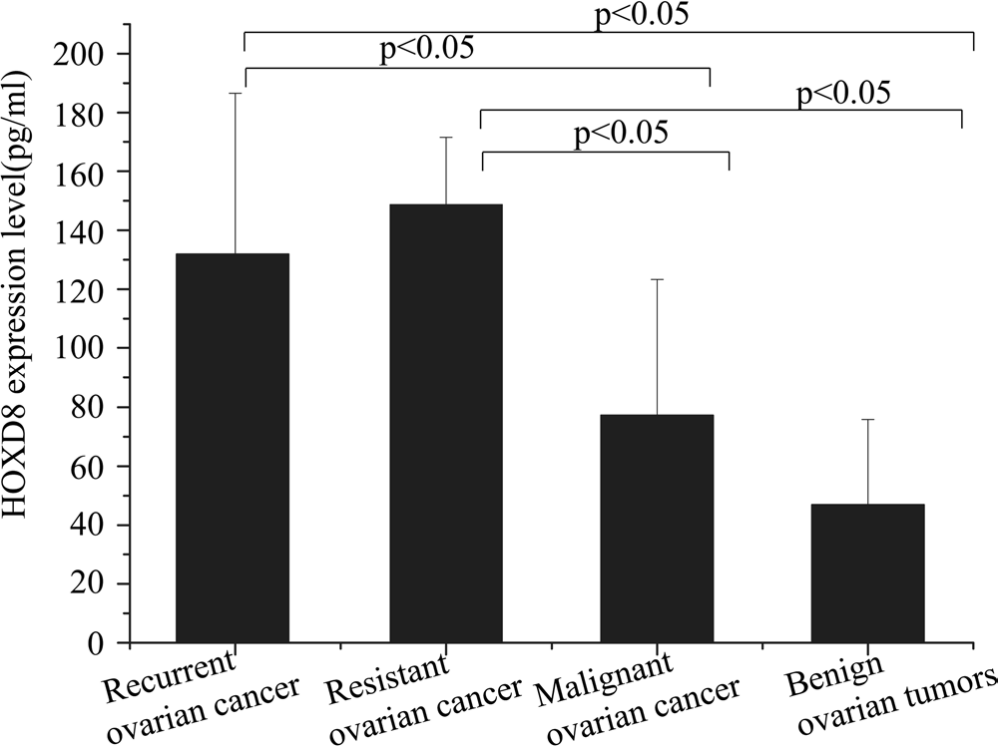
HOXD8 expression levels in human serum. Fifty-two patients were divided in four groups. The patients with recurrent and cisplatin-resistant ovarian cancer had higher expression levels of HOXD8 than patients with primary malignant tumours (*p* = 0.02, *p* = 0.001) or benign tumours (*p* = 0.001, *P* < 0.001). However, the difference between the primary malignant ovarian tumour group and benign ovarian tumour group was not statistically significant (*P* = 0.1).

According to the RECIST 1.1 criteria, 52 participants were enrolled in this survey, including 8 with recurrent ovarian cancer, 10 with cisplatin-resistant ovarian cancer, 20 with primary cisplatin-sensitive malignant ovarian tumours and 14 benign ovarian tumours. Clinicopathological characteristics of patients with different ovarian diseases were show in Table 2. The protein expression levels of HOXD8 in each group were 131.92 ± 54.60 pg/ml, 148.69 ± 22.91 pg/m, 77.31 ± 45.98 pg/ml, and 46.90 ± 29.01 pg/ml, respectively. The patients with recurrent ovarian cancer had higher expression of HOXD8 than patients with primary malignant tumours (*p* = 0.02) or benign tumours (*p* = 0.001). Similary, the same results were observed in the patients with cisplatin-resistant ovarian cancer when compared with primary malignant tumours (*p *= 0.001) or benign tumours (*p* < 0.001). However, the difference between the primary malignant ovarian tumour group and benign ovarian tumour group was not statistically significant (*p* = 0.1) (Fig. 8).

**Table 2:** The clinicopathological characteristics and expresion of HOXD8 in patients with different ovarian diseases (N = 52).

	Recurrent* ovarian cancer (N = 8)		cisplatin-resistant^#^ ovarian cancer (N = 10)		Malignant ovarian tumours (N = 20)		Benign Ovarian tumours (N = 14)	
	N(%)	HOXD8	N(%)	HOXD8	N(%)	HOXD8	N(%)	HOXD8
FIGO stage								
I	4 (50.00)	128.10 ± 53.13	1 (12.50)	133.38	8 (40.00)	51.19 ± 2.51	0	
II	1 (12.50)	43.75	1 (12.50)	133.388	2 (10.00)		0	
III	1 (11.11)	166.41 ± 22.37	7 (70.00)	146.96 ± 20.03	8 (40.00)	110.95 ± 28.35	0	
IV	0		0		2 (10.00)	47.25 ± 3.52	0	
Histology								
serous	5 (62.50)	144.13 ± 58.50	8 (80.00)	145.26 ± 19.16	8 (40.00)	65.03 ± 21.21	2 (14.29)	82.09 ± 26.92
Non-serous	3 (37.50)	111.58 ± 50.96	2 (20.00)	162.39 ± 41.03	12 (60.00)	87.14 ± 25.64	12 (85.71)	34.75 ± 14.36
Lesion location								
Unilateral	4 (50.00)	94.63 ± 53.68	3 (30.00)	144.80 ± 15.51	12 (60.00)	83.95 ± 22.81	13 (0.93)	42.16 ± 12.43
bilateral	4 (50.00)	169.22 ± 19.11	7 (70.00)	150.36 ± 26.38	8 (40.00)	64.03 ± 25.60	1 (0.07)	33.25

*The patients with recurrent ovarian cancer, the median disease-free survival was 20.4 months, range from 12–24 months and the median overall survival was 36.4 months, range from 24–62 months).

^#^The patients with cisplatin-resistant ovarian cancer, the median progression free survival was 4.2 month, range from 3–5 months and the median overall survival was 12 months, range from 9–28 months.

## Discussion

Metastasis and recurrence of malignant tumours are important factors in the survival rate of patients with ovarian cancer. Few studies have examined the potential transcription factor simultaneously associated with cisplatin-resistance and metastasis in ovarian cancer. Transcription factors bind to the promoter of a gene to facilitate the binding of RNA polymerase and initiate the expression of said gene. The analysis of transcription factor activity can combine gene and protein functions and determine functional genome information at the transcriptional level. The method commonly applied to study transcription factors is the electrophoretic mobility shift assay (EMSA)^16^, but it can only determine the activity of one transcription factor at a time. The traditional cDNA chip technology^17–19^ cannot be applied in functional studies. Moreover, it is also limited to measuring the activity of one transcription factor at a time. The variability in transcription factor function among tumour cells of different metastatic potential is still lacking systematic research^20^. However, much of this information can be gathered by a transcription factor chip assay, which has high efficiency and sensitivity, and yields abundant information^21^.

In this study, we used SKOV3 and SKOV3-DDP cell lines and HO-8910 and HO-8910PM cell lines as paired groups serving as *in vitro* research models of drug resistance and metastasis of ovarian cancer. These cell lines were validated in our previous studies^22,23^. From the protein/DNA array assessing a total of 345 candidate transcription factors, activity levels of 43 transcription factors were up-regulated, and 31 were down-regulated in the SKOV3-DDP cell line. In the HO-8910PM cell line, activity levels of 13 transcription factors were up-regulated, and 18 were down-regulated. We detected 13 common transcription factors in the SKOV3-DDP and HO-8910PM cell lines, with 4 up-regulated (*HOXD8(1), HOXD8(2), RB, RFX1/2/3*) and 9 down-regulated (*SRE*, FKHR*, Angiotensinogen ANG-IRE, Pax2, CD28RC/NF-IL2B, HLF, CPE, CBFB* and *c-Ets-1*). We verified these results through a transcription factor activity ELISA kit. Of these transcription factors, Wilson *et al*.^24^ first associated expression of Ets-1, a transcription factor whose expression often signals poor prognosis in various cancer types with cisplatin resistance. Additionally, Liu X *et al*.^25^ found that *Pax2* was down-regulated by at least 4-fold in 594 ovarian serous cystadenocarcinomas in comparison with eight normal ovaries according to The Cancer Genome Atlas (TCGA). The most notable common transcription factor identified in our array was *HOXD8*, which was up-regulated in two gene loci. Hence, we recognized *HOXD8* as a noteworthy transcription factor. *HOXD8* is one of organizing *HOX* genes clusters. *HOX* genes are transcription factors partly expressed during embryogenesis^26^ and regulate numerous processes, including cellular proliferation, differentiation, angiogenesis, migration, and apoptosis. Given that oncogenesis involves dysregulation of signalling pathways necessary during organogenesis and normal stem cell renewal, it is not surprising that aberrant *HOX* gene expression is found in various malignancies of the breast^15,27^, brain^28^, colon^29^, and lung^30^. Studies using the ovarian cancer cell line SKOV3 investigated the role of *HOX* genes in ovarian cancer tumourigenesis^31^. To date, *HOXD8* has been reported in breast^15,32^, colon^33^, and lung cancer^30^. We postulated that *HOXD8* is a transcription factor associated with cisplatin resistance and metastasis of ovarian cancer. In the present study, to follow up on the protein/DNA array, we performed quantitative RT-PCR and ELISA to detect the expression of HOXD8 mRNA and protein levels, respectively, in SKOV3 and SKOV3-DDP cell lines. Moreover, the expression of *HOXD8* was increased in the serum samples of patients with recurrent ovarian cancer. However, in a previous study^34^, *HOXD8* was significantly down-regulated in serous epithelial ovarian cancers compared to normal ovarian surface epithelium (OSE), whereas *HOXB2, HOXB3, HOXB5, HOXB7*, and *HOXB8* were up-regulated. Similar results were reported by Bowen *et al*.^35^, who observed that *HOXD8* genes were down-regulated in serous epithelial ovarian cancers relative to normal OSE. Bonome *et al*. determined that high-grade serous epithelial ovarian cancer exhibit under-expressed *HOXD8* and *HOXD10* mRNA compared to normal OSE^36^. However, *HOXD8* mRNA up-regulation was associated with decreased disease-free survival (DFS) compared to tumours without altered mRNA expression^37,38^. Thus, HOXD8 tends to demonstrate variable up- or down-regulation in serous epithelial ovarian cancer. This may be because HOXD8 was detected in mRNA or protein different levels. Moreover, it may be better to detect transcription factor activity of HOXD8 rather than mRNA or protein levels. However, at present, there are very few methods to detect transcription factor activity directly in the clinic^39,40^. We will study this subject further in future research.

To verify the screening results from the protein/DNA array, we also performed an ELISA to analyse the transcriptional activities of transcription factors, and no difference was shown in these two methods. A transcription factor analysis chip is an ideal functional analysis tool. Currently the common methods of studying interactions between DNA and protein require a large amount of time and effort^41^. Panomics’ TranSignal^TM^ Protein/DNA Array simplifies the functional analysis of eukaryotic transcription factors. TranSignal Arrays allow profiling of the activities of multiple transcription factors simultaneously. These arrays can be used to study transcription factor activation in a variety of biological processes. This array-based technology is a significant improvement over cumbersome EMSA that only permits the characterization of a single transcription factor at a time^16^.

To date, most of studies investigating *HOXD8* gene expression as a prognostic or predictive biomarker have been either retrospective or have been conducted in small heterogeneous patient cohorts with lung cancer^30^. Powered replication studies in homogeneous cohorts of ovarian cancer patients treated with standard cisplatin-based chemotherapy will enable a better understanding of the prognostic and predictive relevance of *HOXD8* gene expression as a potential biomarker of cisplatin-resistance and metastasis. *HOXD8* gene expression should be further assessed together with other potential biomarkers and epigenetic regulators to improve ovarian cancer diagnosis and treatment.

## Materials and Methods

### Cell lines and cell cultures

Ovarian cancer cell lines SKOV3 and the related cisplatin-resistant SKOV3-DDP^42^, HO-8910 and its metastatic equivalent HO-8910PM^43^, and OVCAR-3 were purchased from the Type Culture Collection Centre of Chinese Academic of Science (Shanghai, China). All cell lines were cultured in Dulbecco’s modified Eagle’s medium (DMEM) (Gibco; Thermo Fisher Scientific, Inc., Waltham, MA, United States), supplemented with 10% fetal bovine serum (Gibco), 1% penicillin and 1% streptomycin (100 IU/ml), in a 37 °C incubator with 5% CO_2_.

### Treatment with cisplatin

Cisplatin was purchased from Sigma Company and prepared in 100% dimethyl sulfoxide (DMSO) (Shanghai, China). Before drug treatment, ovarian cancer cell lines SKOV3 and SKOV3-DDP were seeded in 12-well plates at a density of 1 × 10^6^ cells per well in 1 mg/mL serum-free DMEM medium for 12 h to achieve adherence. Three groups were treated with final concentrations of 2, 5, or 8 µg/ml cisplatin. After 12 h, cellular apoptosis and proliferation analyses were performed.

### Cell apoptosis analysis by flow cytometry

Cisplatin-treated cells from each group (10^7^ cells) were fixed and stained following the manufacturer’s instructions of the Annexin-V-Fluos Staining kit (Roche, Germany). We analysed cell apoptosis using the FACSCanto™ II flow cytometer (BD, United States). Experiments were performed in triplicate.

### Cellular scratch assay

Horizontal migration of cells was assessed by a scratch assay performed according to previous reports^44^ with minor modification. Cells were seeded at a density of 5.0 × 10^5^ cells/well, then imaged at 40 × magnification with an Olympus IX70 inverted fluorescence microscope (Olympus Corporation, Japan) at 0 and 24 h post-scratching. Image-ProExpress C software 5.1 (Olympus Corporation) was used to measure the change in the cell distance between the scratches. The average horizontal migration rate was calculated using the following formula: (width 0 h − width 24 h)/width 0 h × 100.

### Transwell chamber assay

The cellular invasive capacity of the cell lines was determined using a Matrigel invasion chamber assay, as previous reported^45^. Cells were seeded at a density of 5.0 × 10^5^ cells/well. The number of cells on the underside of the filter was determined by counting cells in five random fields from three filters for each treatment at 200× magnification with an inverted microscope (Olympus Corporation).

### Nuclear and cytoplasmic protein extraction

Nuclear and cytoplasmic proteins were extracted from adherent cells at 70–90% confluence according the NuCLEARTM Extraction Kit (Sigma, United States). In summary, the cells were rinsed with fresh phosphate-buffered saline (PBS), the appropriate amounts of reagents were added to the particular packed-cell volume, the cells were lysed, and the cytoplasmic and nucleic proteins were isolated by gradient centrifugation. Protein concentrations were quantitated with a BCA^TM^ assay kit from Pierce (United States).

### Protein/DNA Arrays

Cultures of SKOV3, SKOV3-DDP, HO-8910 and HO-8910PM were screened for transcription factors using the TranSignal Protein/DNA Array (Panomics, United States) following the manufacturer’s protocol. Briefly, proteins were isolated as described above, and nuclear extracts (25 μg) were incubated with the provided biotin-DNA probe mix for 30 minutes. Protein-DNA complexes were isolated by 2% agarose gel electrophoresis. The proteins were eliminated from the complex and the biotin-DNA could hybridize to the membranes with consensus-binding sequences for transcription factors. After this step, the membranes were incubated with streptavidin-alkaline phosphatase conjugate. Signals of the hybridized probes were visualized using the chemiluminescent imaging system provided with the TranSignal Protein/DNA Array kit and exposed to X-ray film^46^. Using the expression in SKOV3 and HO-8910 cells as the benchmark, a two-fold increase or decrease in expression in the SKOV3-DDP/SKOV3 or HO-8910PM/HO-8910 cells was considered a significant change. Experiments were performed in duplicate.

### ELISA based transcription activity assay

The TransAM^TM^ transcription factor activity enzyme-linked immunosorbent assay (ELISA) kit (Active Motif companies, United States) was used to perform an ELISA-based transcription activity assay. Nucleoprotein extraction (10 µg in 10 µL pyrolysis buffer), the positive control (5 µg positive nucleoprotein in 10 µL pyrolysis buffer), or negative control (10 µL pyrolysis buffer) was added to 40 µL buffer and centrifuged at 100 rpm for 1 h at room temperature. The nuclear extract was incubated with an antibody against FKHR, CREB and NF-ĸB P65 (1:1000) at room temperature for 1 h and washed three times. It was then incubated with 100 µL (1:1000) anti-IgG horseradish peroxidase conjugate, and washed three times. The plate was developed, the reaction stopped, and the optical density (OD) measured at 450 nm using a microtiter plate reader (655 nm for reference).

### RNA isolation and RT-PCR

Total RNA was isolated according to the Trizol kit protocol (Invitrogen, United States). The quantity and quality of the mRNA was assessed using the DNA Counter (Bio-RAD, USA). Only samples with an OD 260/280 ratio exceeding 1.8 were used in the experiments. The Access RT-PCR system (Promega, United States) was used according to the manufacturer’s instructions. Real-time PCR was performed with 2 µL cDNA using the LightCycler® 480 SYBR Green I Master Mix according to the manufacturer’s instructions (Roche, Germany). The programme was as follows: 35 cycles of pre-denaturation at 95 °C for 5 min, denaturation at 95 °C for 30 s, annealing at 61 °C for 30 s, and extension at 72 °C for 60 s, followed by a final extension at 72 °C for 5 minutes. The following primer set for *HOXD8* was synthesized by Takara Biotechnology (Dalian, China): sense, 5′-CCT GAC TGT AAA TCG TCC AGT GGT A-3′; and anti-sense 5′-AGT TTG GAA GCG ACT GTA GGT TTG-3′. The *GAPDH* primer set was purchased from Takara (HA067812, GenBank: NM-002046). The PCR products were 147 bp in length for *HOXD8* and 226 bp for *GADPH*. The relative mRNA levels were calculated using the comparative cycle threshold (Cq) method (ΔΔ Cq)^47^.

### Quantitative protein ELISA

ELISAs were performed as described previously, with minor modifications. After the reaction, the OD of each well was measured within 5 minutes at 450 nm alongside an enzyme standard. The “Curve Expert 1.3” was used to make a standard curve to determine sample concentration.

### Lentivirus-mediated vector construction and infection

Lentivirus mediated overexpressing HOXD8 vector or control vector were constructed and purchased from Genechem Company (GCD0161567, Shanghai, China). The OVCAR-3 and HO-8910 cells were infected with lentivirus mediated control vector (NC group) or overexpressing HOXD8 vector (OV group) at multiplicities of infection (MOIs) of 20 (low MOI) and 80 (high MOI) as our previous reported^48^. After infection for 72 h, GFP expression was detected to calculate the infection efficiency, and cells were harvested in fifth day after infection. Real-time PCR and protein ELSIA were performed to determine the highest expression of HOXD8 and used for subsequent experiments. The OVCAR-3 and HO-8910 cells were infected with lentiviruses containing control vector were named as OVCAR-3-NC and HO-8910-NC, while cells were infected with lentivirus-mediated overexpressing HOXD8 were name as OVCAR-3-OV and HO-8910-OV.

### MTT assay

The OVCAR-3-NC, HO-9010-NC, OVCAR-3-OV and HO-8910-OVcells were implanted in 96-well plates with 1500 cells per well and treated with final concentrations of 0, 2, 5, or 8 mg/L cisplatin, respectively. Cellular proliferation was measured by means of MTT assay at 0, 24, 48, 72, 96 h, and their absorbance values were measured at 490 nm wavelength with a spectrophotometric plate reader.

### Population information

All participants provided written informed consent, and all study protocols were approved by the ethics committee of Fujian Maternity and Children Health Hospital (Fujian, China) (approval number 2013004). All methods of data collection and analysis were performed in accordance with relevant guidelines and regulations with appropriate quality control. Serum was collected before the time of surgery. According to the RECIST 1.1 criteria, the study involved 8 cases of recurrent ovarian cancer (the median disease-free survival was 20.4 months, range from 12–24 months), 10 with cisplatin-resistant ovarian cancer (median progression free survival was 4.2 month, range from 3–5 months), 20 with primary malignant ovarian tumours and 14 benign ovarian tumours. The ages of each group were 39.88 ± 12.97, 47.70 ± 9.03, 43.44 ± 16.48 and 48.25 ± 13.91 years, respectively. These patients were diagnosed based on pathology after surgery. ELISA was applied to detect the HOXD8 expression level in human serum.

### Statistical analysis

Experimental data were processed by SPSS software 17.0 for Windows (SPSS Inc., Chicago, IL, United States) and expressed as the mean ± SD. Wherever appropriate, the data were also subjected to unpaired two-tailed Student’s *t* tests or one-way ANOVA. Differences were significant if *p* < 0.05.

## Data Availability

All data generated or analysed during this study are included in this published article.
